# Supplementary material for: Sialic acid mediated mechanical activation of β2 adrenergic receptors by bacterial pili
Source: Nat Commun. 2019 Oct 18;10:4752. doi: 10.1038/s41467-019-12685-6 (PMC6800425; doi:10.1038/s41467-019-12685-6)
Supplement: Supplementary file 1 — Supplementary Information [file 41467_2019_12685_MOESM1_ESM.pdf]

Supplementary Information

Sialic acid mediated mechanical activation of β2 adrenergic receptors by bacterial pili

Virion et al.

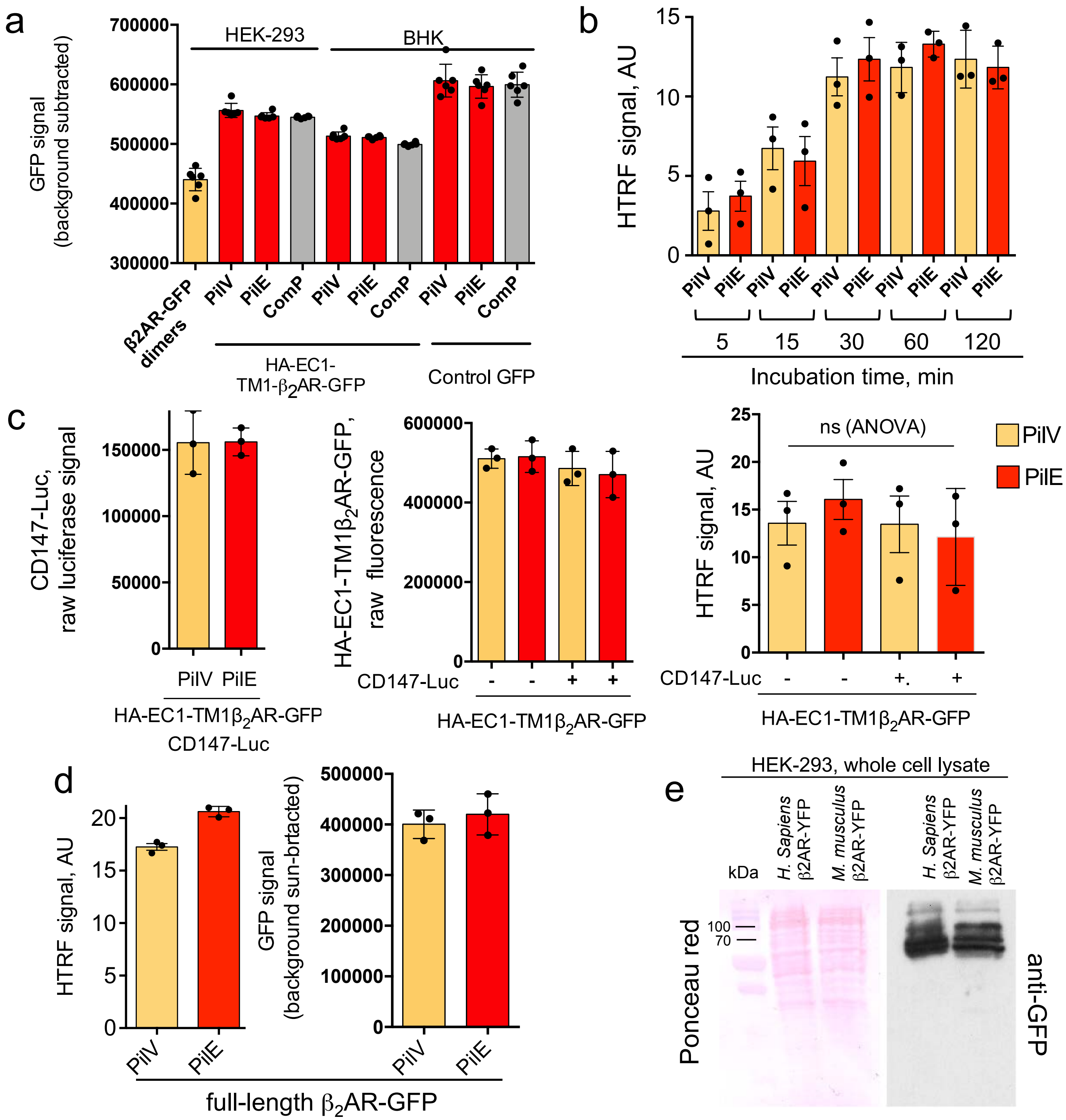

Supplementary Figure 1. Control experiments related to Fig. 1. a) Raw specific (background-subtracted) fluorescence signals corresponding to the experiment in Fig. 1b. **b**) Kinetic experiment conducted in the same conditions as in Fig. 1b to determine the optimal incubation time with HTRF-antibodies. **c**) HTRF in the presence of co-expressed CD147-Luc and corresponding controls. PilV yellow bars, PilE red bars. **d**) HTRF experiment as in Fig. 1b on HEK-293 cells expressing full-length β_2AR-GFP instead of HA-EC1-TM1β_2AR-GFP. **e**) WB experiments to compare the expression of human and mouse β_2AR-GFP in HEK-293 cells. Since the transfection rate was much lower in mouse C166 cells (about 5%, versus >90% in HEK-293), signaling events were counted under the microscope by specifically examining bacterial colonies above fluorescent cells (expressing the β_2AR-GFP). Note that in the absence of exogenous CEACAM-1 expression meningococci do not adhere to C166 cells. The experiments in **c** and **d** are representative of 3 independent experiments giving the same result but at different levels of exogenously expressed proteins. In all panels bars correspond to SEM

Species	Receptor	N-terminus sequence	Nm-induced signaling *in vitro*
Homo s.	β_2AR	MGQPGNGSAFLLAPNGSHAPDHDVTQQ	+++
Mus m.	β_2AR	MGPHGNDSDFLLAPNGSRAPDHDVTQE	+++/– (*)
Pan t.	β_2AR	MGQPGNGSAFLLAPNGSHAPDHDVTQE	ND
Macaca m.	β_2AR	MGQPGNGSAFLLAPNGSHAPDHDVTQE	ND
Rattus n.	β_2AR	MEPHGNDSDFLLAPNGSRAPGHDVTQE	ND
Bovis t.	β_2AR	MGQPGNRSVFLLAPNASHAPDQNVTLE	ND
Sus s.	β_2AR	MGQPGNRSVFLLAPNGSHAPDQDVPQE	ND
Canis f.	β_2AR	MGQPGNRSVFLLAPNGSHAPDQGDSQE	ND
Homo s.	β_2AR-N6A	MGQPGAGSAFLLAPNGSHAPDHDVTQQ	–
Homo s.	β_2AR-N15A	MGQPGNGSAFLLAPAGSHAPDHDVTQQ	–
Rattus n.	AT1R	MILNSSTEDGIKRIQDDCPKAGRHS	–
Rattus n.	HA-AT1R-2NglyHuβ_2AR	MYPYDVPDYAALNGSAEDGIKNGSHDCPKAGRHS	+++

Supplementary Figure 2. Alignment of mammal β_2AR N-terminal amino-acid sequences.

The shown sequences correspond to the region of the human β_2AR, which confers the capacity of transducing signalling events in response to meningococcus, once transposed into the angiotensin AT1R. Boxed sequences: mutated β2AR lacking either N-glycan chain (β_2AR-N6A and β_2AR-N15A), angiotensin AT1R and HA-tagged AT1R containing the β_2AR N-glycosylation consensus sequences (HA-AT1R-2NglyHuβ_2AR) at the same distance as in the β_2AR. Residues in red indicate variation respective to the amino-acid residue present at the same position in the human β_2AR sequence. Residues in green correspond to the HA tag. Asparagine residues where glycan chains are branched are highlighted in yellow. Presence (+++) or absence (-) of meningococcal-induced signalling in an *in vitro* assay for ezrin translocation to *N. meningitidis* colonies growing at the surface of human cells expressing exogenous β_2ARs and β-arrestins (as in Fig. 1d and 2 and described in ref. [9]); ND: not determined. The asterisk highlights the divergent signal in human (positive) and mouse (negative) cells in the assay above.

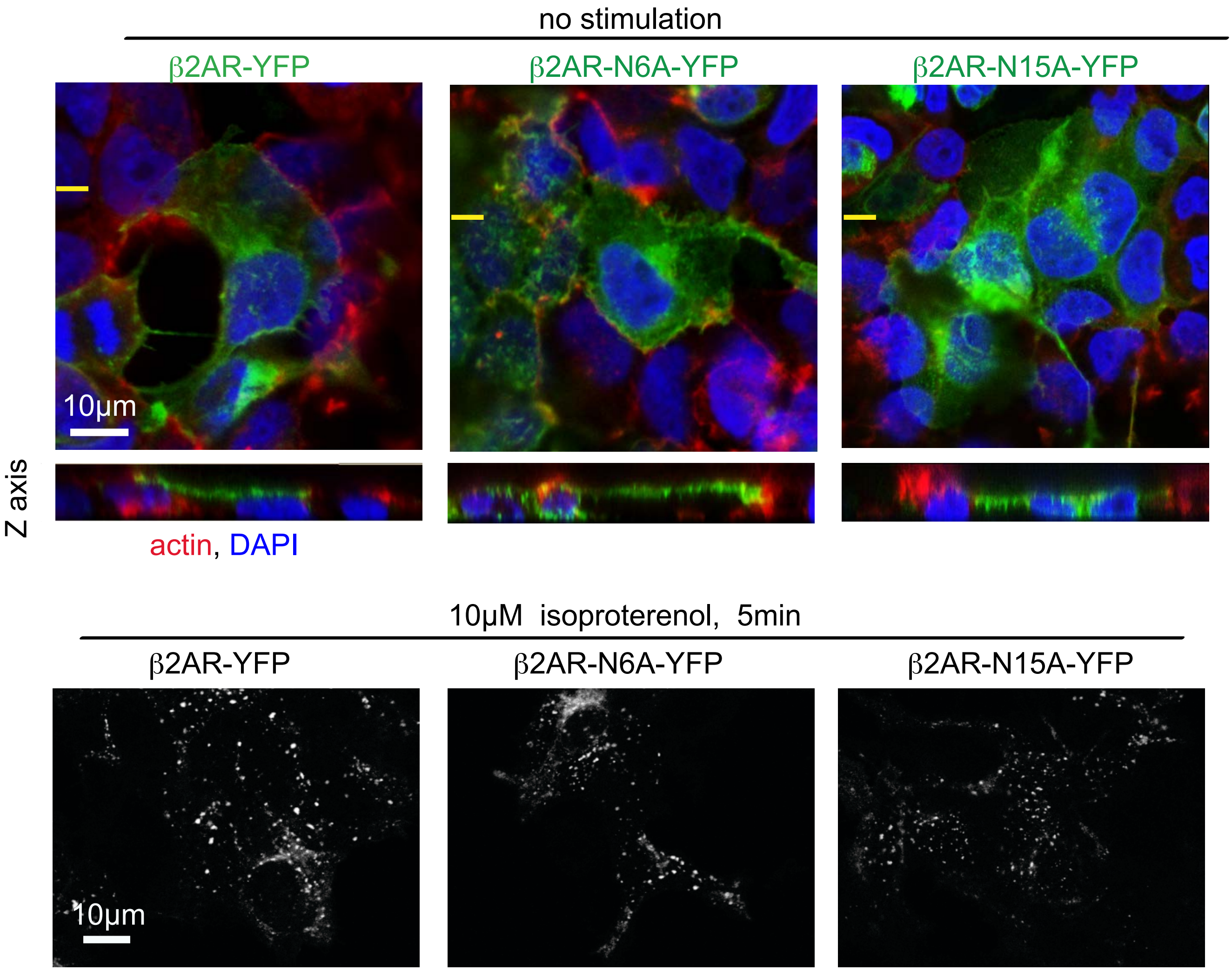

Supplementary Figure 3. β_2AR mutants lacking either N-glycosylation site are normally processed to the cell surface and sensitive to receptor agonist.
HEK-293 cells were transfected with plasmids coding for YFP-tagged wild type β_2AR (β_2AR-YFP) or for receptor mutants, in which the asparagine residue at position 6 (β_2AR-N6A-YFP) or 15 (β_2AR-N15A-YFP) were replaced by an alanine residue. Top: Resting cells were fixed and submitted to fluorescence confocal microscope analysis. Z series (at the level of the yellow bar) show that in all cases receptors were mostly localised to the cell surface. Bottom. Cells were incubated with the β-adrenergic agonist isoproterenol for 5 min at room temperature before fixation. In all cases this treatment induced the expected β-arrestin-dependent redistribution of the β_2AR into spots corresponding to clathrin-coated vesicles and early endosomes, demonstrating that the receptor mutants remain sensitive to the agonist and can recruit β-arrestin.

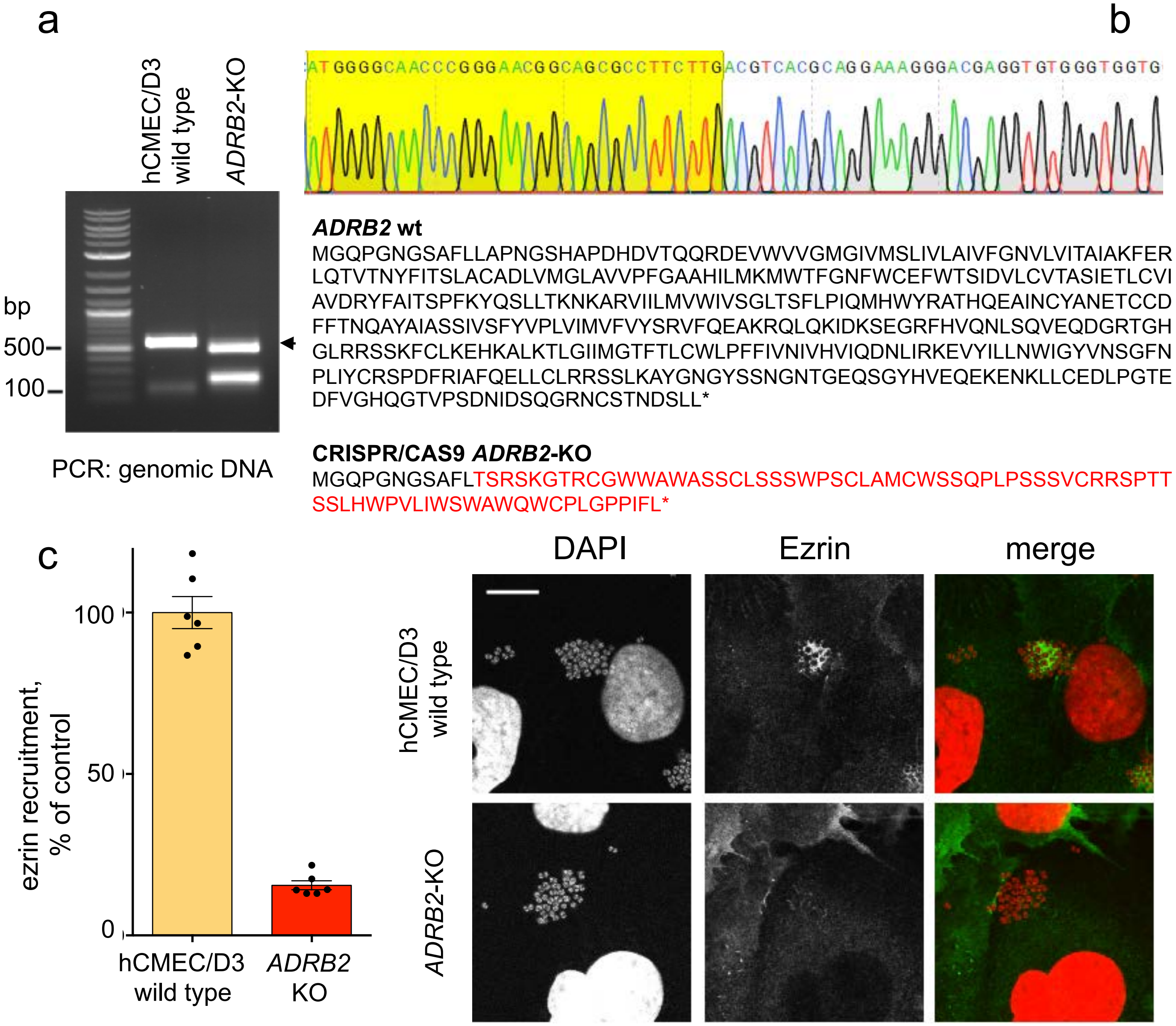

Supplementary Figure 4. Deletion of *ADRB2 in* hCMEC/D3 endothelial cells markedly inhibits meningococcal signaling.

a) Bi-allelic *Adrb2* deletion in hCMEC/D3 human endothelial cells. Intron-less genomic DNA was prepared from hCMEC/D3 wild type and hCMEC/D3 Knock-Out cells. A region including the first 300 bp of *Adrb2* was amplified by PCR using Hu-ADRB2_Fwd and Rv primers (Suppl. Table 2). These primers amplify a 548bp fragment from wild type genomic DNA (arrow). PCR products were sequenced using the same primers. **b**) Top: chromatogram of the genomic DNA sequence of the *ADRB2*-KO clone. The wt sequence preceding the deletion is highlighted in yellow. Bottom: deduced amino-acid sequences of wt and *ADRB2*-KO clones. The deletion resulted in a frame shift (in red) with early termination (asterisk). **(c)** Wt and *Adrb2*-KO cells, which both express endogenous CEACAM-1, were infected with the SiaD-Opa+ 2C4.3 strain expressing the Opa secondary adhesin, to avoid any potential adhesion defect caused by the lack of β2AR expression. Cells were washed and fixed and stained with Alexa-conjugated anti-ezrin antibodies. Dapi was used to stain nuclei and bacterial DNA before analysis with a fluorescence microscope. Ezrin recruitment at the site of bacteria adhesion was expressed as % of control ± SEM. Data, corresponding to two independent experiments in triplicate were analyzed by student *t* test $p<0.0001$. Right panel: Representative images. Bar: 10µm.

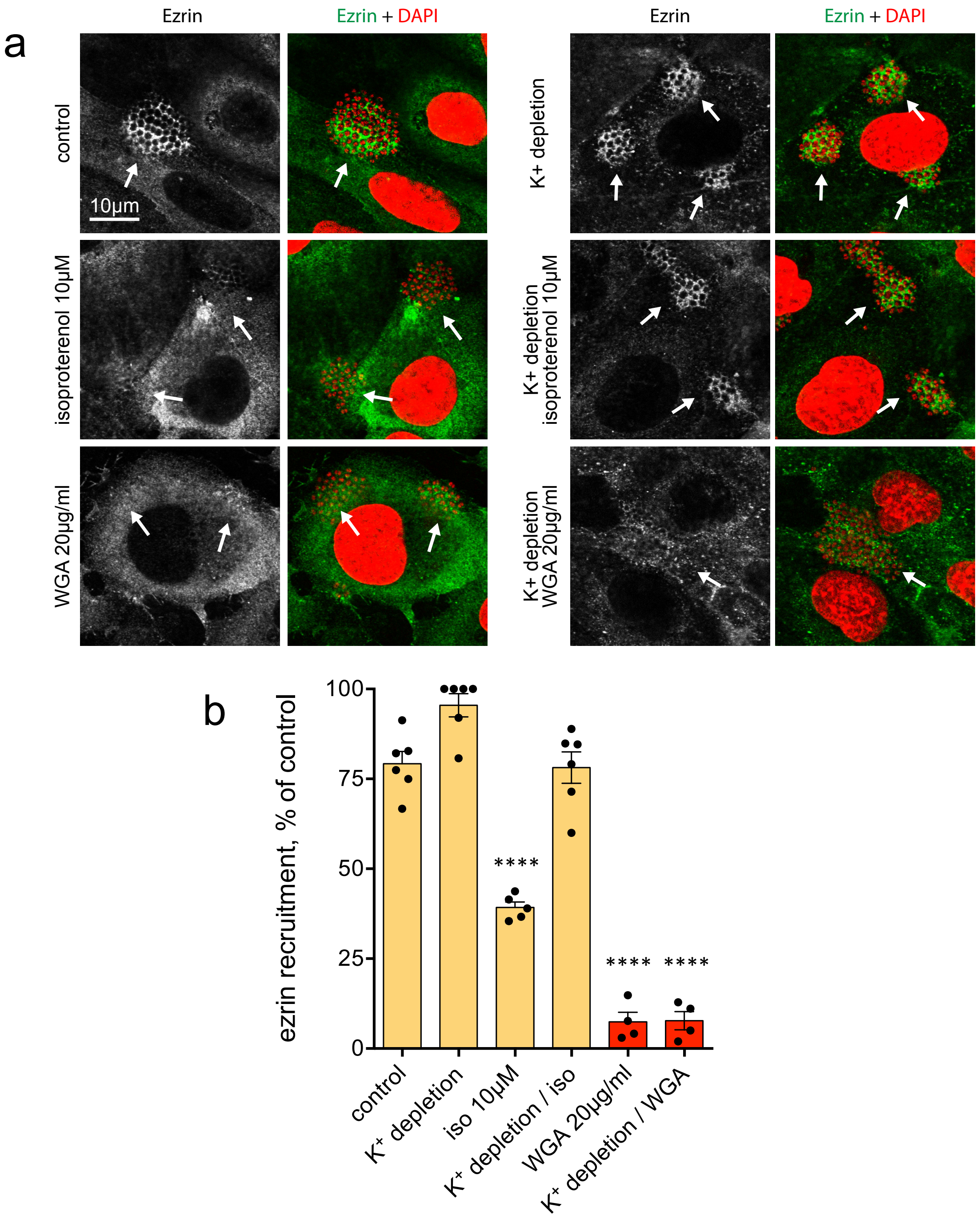

Supplementary Figure 5. Inhibition of *N. meningitidis* signalling by WGA is unrelated to β_2AR endocytosis. Human endothelial hCMEC/D3 cells in normal (control) or K^+-depleted (to inhibit receptor endocytosis) medium, were pre-incubated with 20 µg/ml WGA or 10 µM isoproterenol (iso) for 1 hour, before meningococcal infection. Cells were then processed and analyzed as in Fig. 3. **a**) representative images; arrows indicate bacterial colonies. **b**) quantitative analysis by ANOVA with Dunnett's multiple comparisons test. **** p<0.0001 control vs. the indicated condition. By inhibiting β_2AR endocytosis, K^+ depletion abolished the inhibition of meningococcus-promoted signalling induced by isoproterenol (see [9]). K^+ depletion had no effect on the inhibition of *N. meningitidis* signalling by WGA, consistent with the hypothesis that WGA effect does not involve receptor endocytosis.

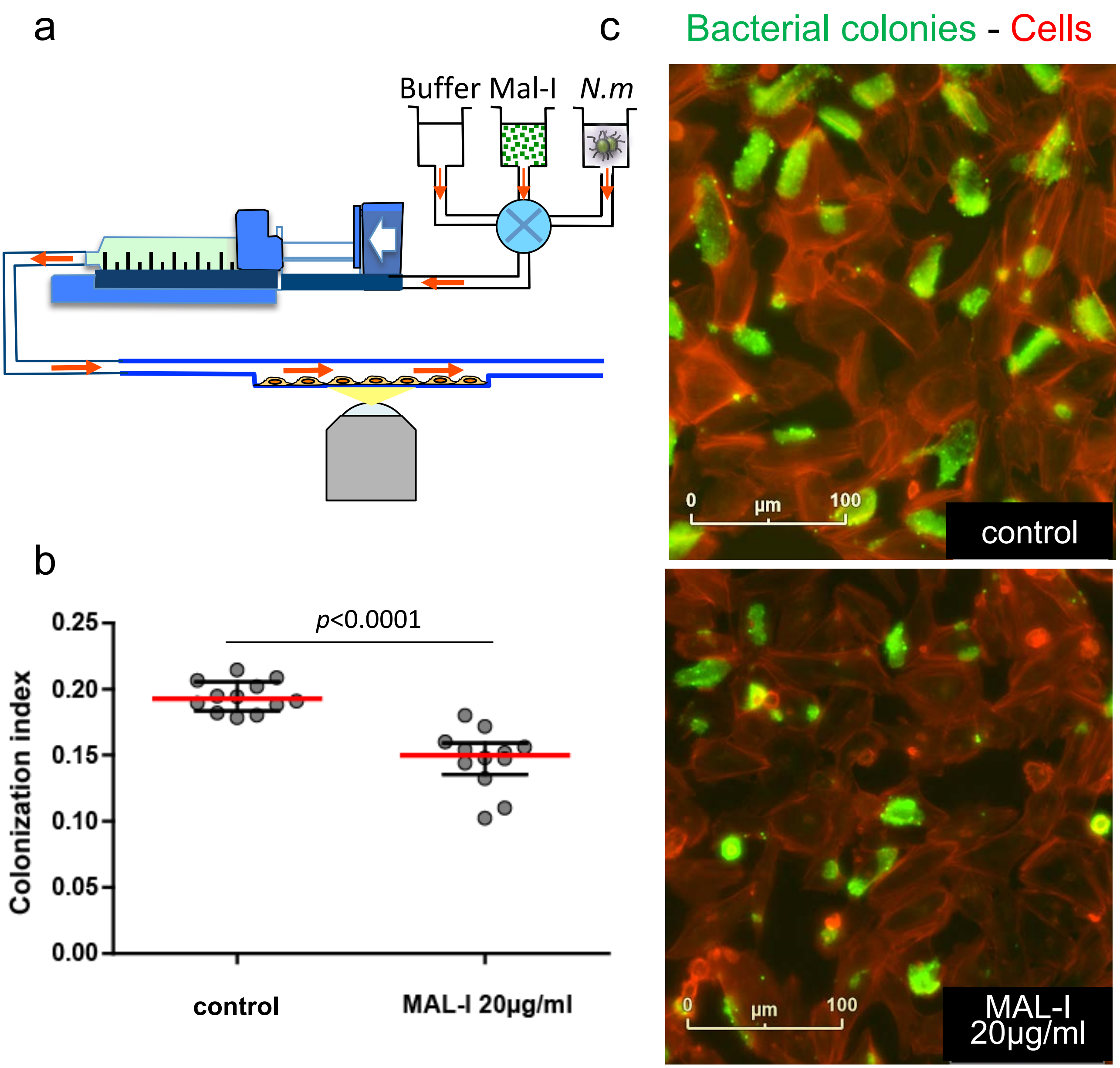

Supplementary Figure 6. In vitro meningococcal colonization of hCMEC/D3 cells under shear stress is also inhibited by sialic acid-binding lectins.
a) Diagram of the experimental design. Wild type endothelial hCMEC/D3 cells grown in Ibidi™ chambers, pretreated or not (buffer alone) with lectin MAL-I (20µg/ml), were infected under stopped flow and then submitted to a laminar shear stress of 0.4 dynes/cm2 generated by an electric pump for 3 hours in buffer alone. After cell fixation, bacteria were stained using the anti-2C43 rabbit antibody and cells stained with phalloidin. Four Images (900x600µm) were acquired per chamber using the Incucyte S3 system**.** **b)** Colonization was quantified using the Incucyte S3 software and expressed as the median colonization index (total surface of bacteria over total surface of cells, both expressed in µm^2) with interquartile range. Statistical significance was assessed by Mann Whitney U test**.** Bars: SEM **c**) Representative fields.

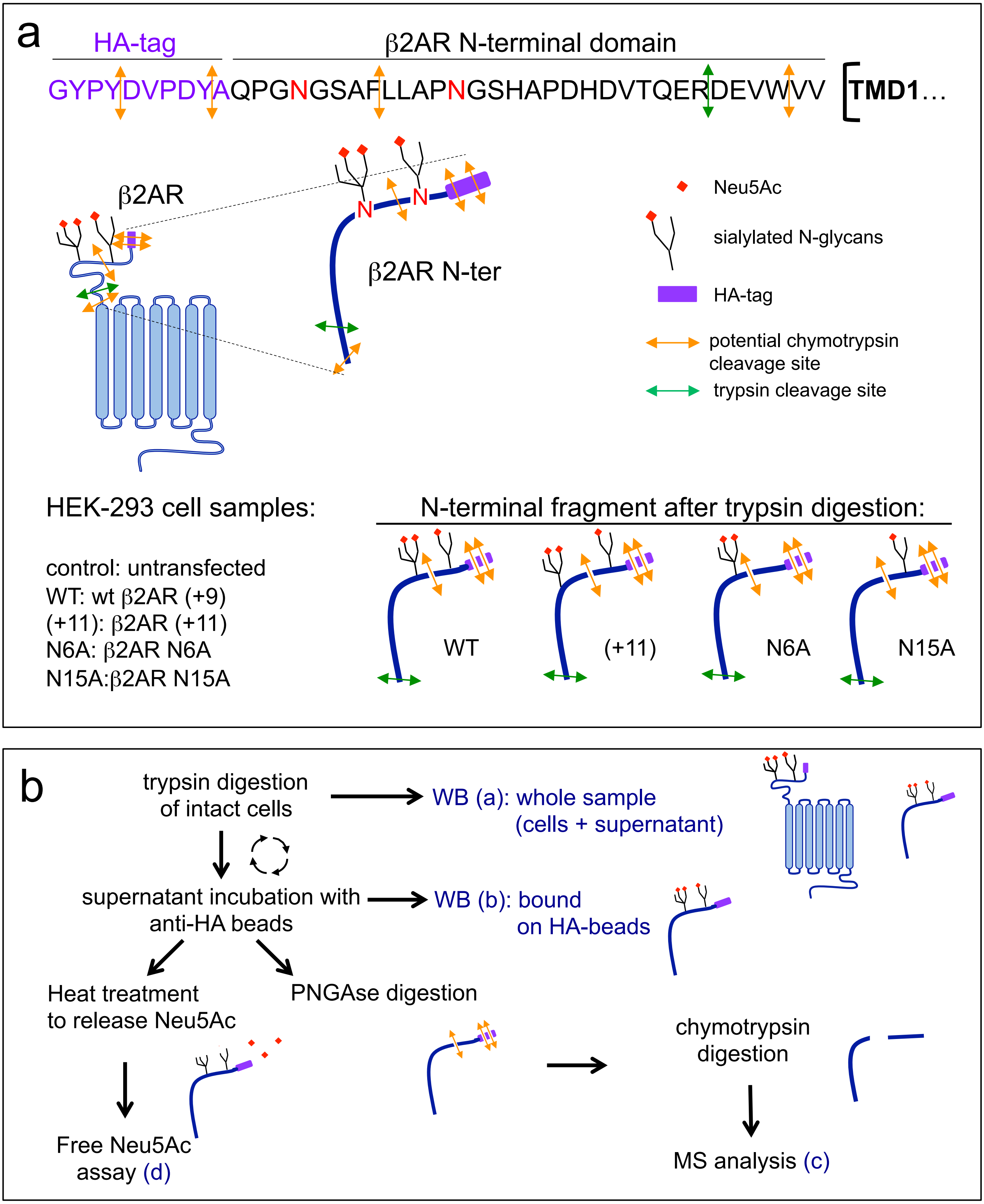

Supplementary Figure 7. Sialylation of β2AR N-glycan chains: procedure outline
a) Cartoon of wt and mutant β2AR N-termini released after trypsin cleavage. **b**) Diagram of the experimental approaches used to produce the data shown in Fig. 4

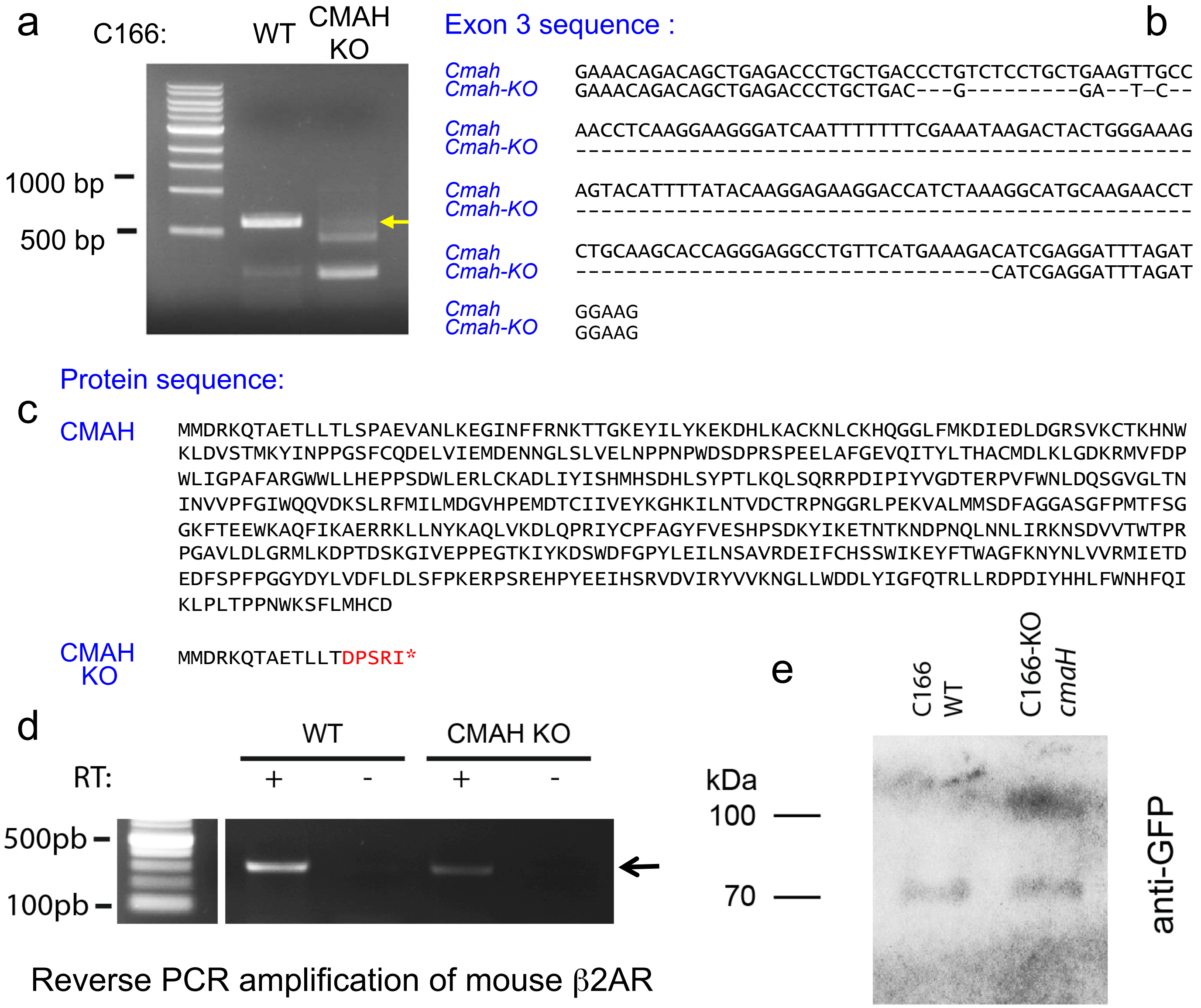

Supplementary Fig. 8. Bi-allelic *Cmah* deletion in C166 mouse endothelial cells. (a) Genomic DNA was prepared from C166 wild type and CMAH Knock-Out cells (see "CRISPR/Cas9 genome editing" in Methods section). A region including *cmah* Exon 3 was amplified by PCR using the CMAH-E3_Fwd and Rv primers (Suppl. Table 2). These primers amplify a 597bp fragment from wild type genomic DNA (arrow). The absence of such a fragment after PCR from the CMAH-KO C166 clone confirms the bi-allelic deletion of part of the Exon 3. **(b)** PCR products were sequenced using CMAH seq Fwd and Rv primers (Suppl. Table 2). The wild type and mutated sequences were aligned using CLUSTALW2. **(c)** Deduced amino-acid sequence in the CMAH-KO cone. The mutation resulted in a frame shift (in red) with early termination at position 19 (marked by the asterisk). (**d**) Both wt and *CMAH*-KO C166 express endogenous β2AR. Reverse Transcription (RT: +) was carried out with 400 ng of RNA from these cells using M-MLV Reverse Transcriptase (Promega). Negative controls (RT: -) without M-MLV Reverse Transcriptase were performed in parallel. PCR were performed using the mus-ADRB2_Fwd and Rv primers (Suppl. Table 2). (**e**) Wt and *cmaH*-KO cells were transfected with a plasmid coding for β2AR-YFP. The % of transfected cells was equivalent, determined by counting "green cells" per field under the microscope. Cell lysates were incubated with Neu5Ac-selective WGA-beads. After washings, beads were resuspended in Laemmli buffer, samples separated by PAGE, blotted and probed with anti-GFP antibodies.

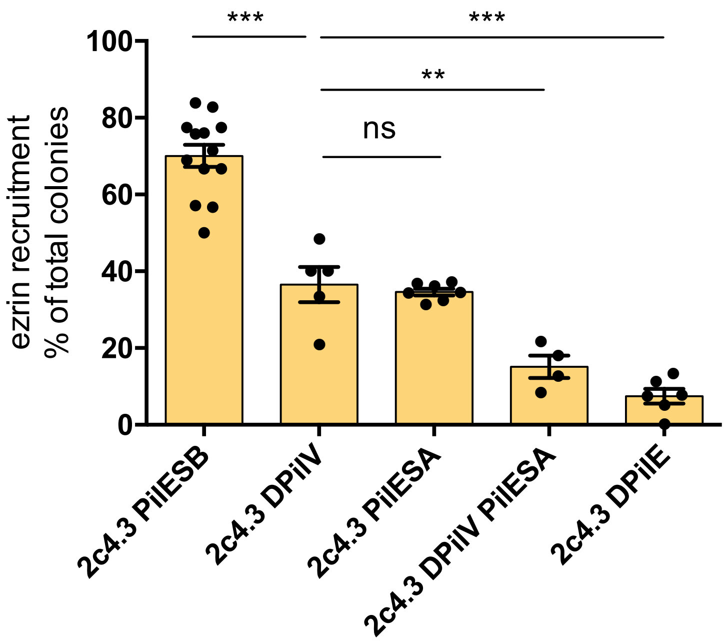

Supplementary Fig. 9. Synergy between pilins for maximal bacterial-induced signaling in host cells.

The ezrin-recruitment assay in the reconstituted HEK-293 system (described in Fig. 1d) was conducted with the indicated meningococcus strains. Some bacterial strains were selected expressing either variant of PilE pilin (namely, PilESA and PilESB), or deleted of PilV gene (ΔPilV) in the context of PilESA or PilESB variants, or deleted of PilE gene (ΔPilE). In the last case, due to the absence of the major PilE pilin, bacterial pili are totally absent. Either PilV gene deletion or PilESA variant caused a significant decrease of ezrin recruitment compared to standard PilESB strain. When these two modifications were combined the inhibition of ezrin recruitment was further enhanced (compare ΔPilV-PilESA with ΔPilV-PilESB). Bars: SEM; ** $p<0.01$; *** $p<0.001$.

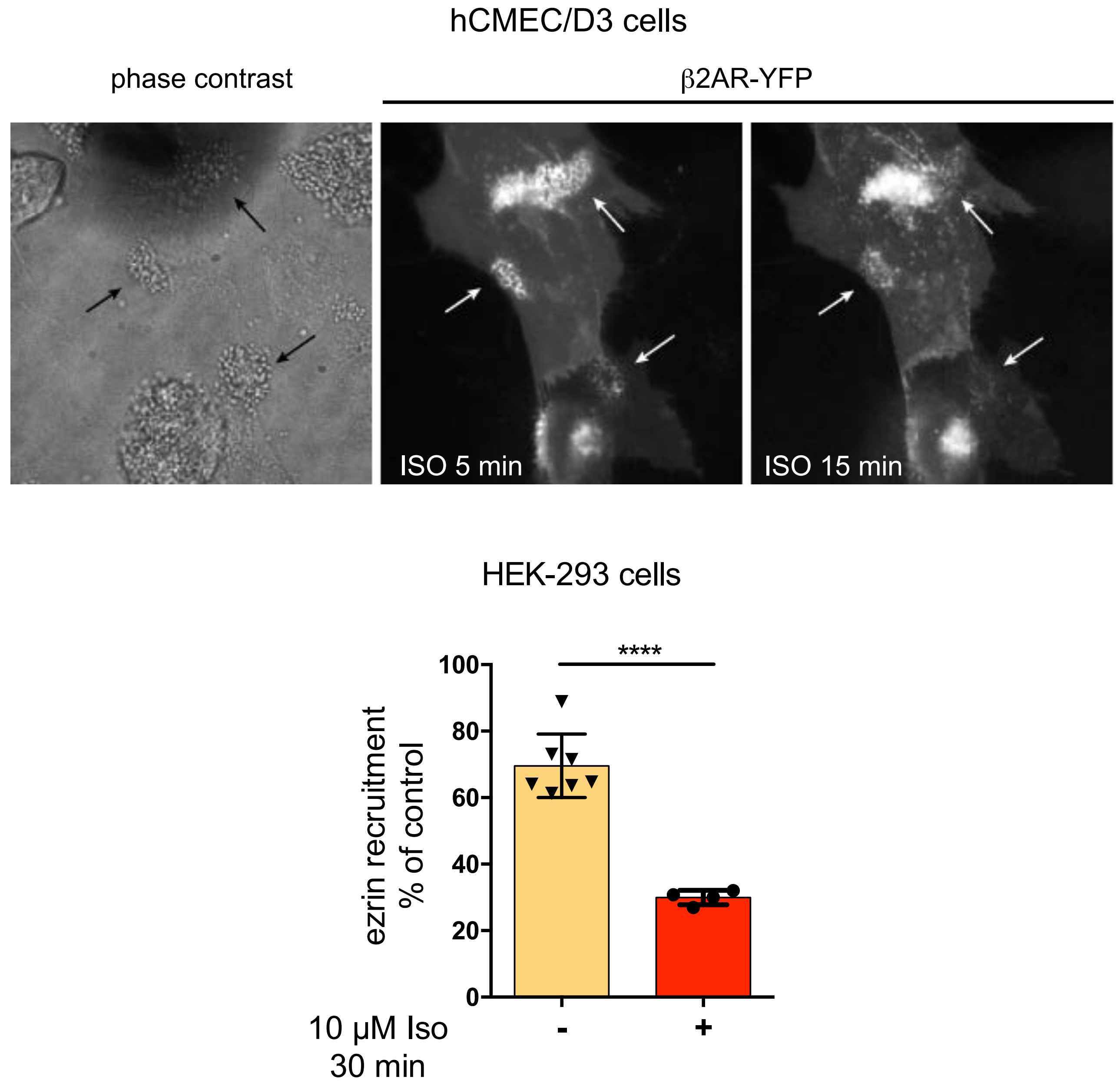

Supplementary Fig. 10. Cell infection in the presence of Isoproterenol
hCEMEDC/D3 cells expressing exogenous β2AR-YFP (Top) or HEK-293 cells reconstituted with β2AR, β−arrs and CEACAM, infected with meningococcus in the presence or absence of 10 µM Isoproterenol (Iso) for the indicated times. β2AR-YFP was followed in real time under the microscope. HEK-293 cells were then washed, fixed and stained with Alexa-conjugated anti-ezrin antibodies. Ezrin recruitment was determined as in Fig. 1d. 15 min after infection in the presence of Iso, the β2AR accumulated in dots, likely corresponding to endosomes, while the amount of the receptor beneath the colonies (arrows) decreased. in the HEK-293 reconstitution system, after 30 min incubation with ISO ezrin recruitment was reduced compared to control cells. Bars: SEM; $p<0.0001$

Supplementary Table 1: Lectins used in this work

Lectins name	Target	Abbreviations
Concanavalin A	D-mannose or D-glucose	ConA
Glycine max (soybean) agglutinin	α/β N-acetylgalactosamine	SBA
Triticum vulgaris (wheat germ) agglutinin	sialic acid and poly N-acetylglucosamine	WGA
Succinylated wheat germ agglutinin	N-acetylglucosamine oligomers	sWGA
Dolichos biflorus agglutinin	α-N-acetylgalactosamine	DBA
Ulex europaeus agglutinin 1	α-L-Fucose ; anti-H blood group specificity	UEA 1
Ricinus communis agglutinin	β-D-galactosyl residues	RCA_{120}
Arachis hypogaea (peanut) agglutinin	galactosyl (β-1,3) N-acetylgalactosamine	PNA
Griffonia simplicifolia lectin I	terminal α-D-galactose	GSL I
Pisum sativum agglutinin	terminal α-D-mannose	PSA
Lens culinaris agglutinin	α-D-mannosyl and α-D-glucosyl residues	LCA
Phaseolus vulgaris Erythroagglutinin	red cell agglutination	PHA-E
Phaseolus vulgaris Leucoagglutinin	lymphocyte agglutination	PHA-l
Sophora japonica agglutinin	β-N-acetylgalactosamine	SJA
Succinylated wheat germ agglutinin	N-acetylglucosamine oligomers	sWGA
Griffonia simplicifolia lectin II	α/β N-acetylglucosamine	GSL II
Datura Stramonium lectin	(β-1,4) linked N-acetyl-D-glucosamine oligomers	DSL
Erythrina cristagalli lectin	D-galactose and D-galactosides	ECL
Jacalin	galactosyl (β-1,3) N-acetylgalactosamine (mono or di sialylated)	Jacalin
Lycopersicon esculentum (tomato) lectin	N-acetyl-β-D-glucosamine and N-acetyl-D-lactosamine oligomers	LEL
Solanum tuberosum lectin	N-acetyl-β-D-glucosamine and N-acetyl-D-lactosamine oligomers	STL
Vicia villosa agglutinin	N-acetyl-D-galactosamine	VVA
Maackia amurensis lectin	Sialic acid (α2-3) gal (β-1,4) glcNAc	MAL I / MAA
Maackia amurensis lectin II	Sialic acid (α2-3) N-acetyl-galactosamine	MAL II / MAH
Sambucus nigra bark lectin	Sialic acid (α2-6) gal (β-1,4) glcNAc	SNA
Phytolacca americana (pokeweed) lectin	N-acetyl-β-D-glucosamine oligomers	PAL / PWA

Sources: www.vectorslabs.com and www.sigmaaldrich.com

Supplementary Table 2: Primers used in this study

PilV_fw : ggatccCCGGCGCGTCCGC

PilV_Rv: ctcgagGTCGAAGCCGGGGC

PilE_fw : ggatccCGCCCGCGCACAAGTTTCCG

PilE_Rv: ctcgagGCTGGCAGATGAATCATCGC

ComP_fw : ggatccCGAGAAAGCAAAGATAAATGC

ComP_Rv: ctcgagCTTAAATAACTTGCAGTC

β_2AR_Fwd: AGCTCTCGAGATGGGGCCACACGGGAACGAC

β_2AR_Rv: CCGCAAGCTTCAGTGGCGAGTCATTTGTACTACAG

Hu-ADRB2_Fwd: CAAGCTGAGTGTGCAGGACGAGTC

Hu-ADRB2_Rv: AGGCCAGTGAAGTGATGAAG

mus-ADRB2_Fwd: TCGAGACCCTGTGCGTGATTG;

mus-ADRB2_Rv: CTGGAAGACCCGGG AATAGAC

CMAH-E3_Fwd: AAAGGTGGGGAGAAAGGGTA

CMAH-E3_Rv: CTATTGGGGTGTTTGGGTTG

CMAH_seq Fwd: TGCATGCTCCTCTGGTAATG

CMAH_seq Rv: ACTGGCAAGAGAGCAAAGGA
