# Supplementary material for: Sialic acid mediated mechanical activation of β2 adrenergic receptors by bacterial pili
Source: Nat Commun. 2019 Oct 18;10:4752. doi: 10.1038/s41467-019-12685-6 (PMC6800425; doi:10.1038/s41467-019-12685-6)
Supplement: Supplementary file 2 — Reporting Summary [file 41467_2019_12685_MOESM2_ESM.pdf]

nature research

Corresponding author(s): Stefano Marullo

Reporting Summary

Nature Research wishes to improve the reproducibility of the work that we publish. This form provides structure for consistency and transparency in reporting. For further information on Nature Research policies, see Authors & Referees and the Editorial Policy Checklist.

Statistical parameters

When statistical analyses are reported, confirm that the following items are present in the relevant location (e.g. figure legend, table legend, main text, or Methods section).

n/a	Confirmed	
☐	☒	The exact sample size (*n*) for each experimental group/condition, given as a discrete number and unit of measurement
☐	☒	An indication of whether measurements were taken from distinct samples or whether the same sample was measured repeatedly
☐	☒	The statistical test(s) used AND whether they are one- or two-sided<br>*Only common tests should be described solely by name; describe more complex techniques in the Methods section.*
☒	☐	A description of all covariates tested
☐	☒	A description of any assumptions or corrections, such as tests of normality and adjustment for multiple comparisons
☐	☒	A full description of the statistics including central tendency (e.g. means) or other basic estimates (e.g. regression coefficient) AND variation (e.g. standard deviation) or associated estimates of uncertainty (e.g. confidence intervals)
☐	☒	For null hypothesis testing, the test statistic (e.g. *F*, *t*, *r*) with confidence intervals, effect sizes, degrees of freedom and *P* value noted<br>*Give P values as exact values whenever suitable.*
☒	☐	For Bayesian analysis, information on the choice of priors and Markov chain Monte Carlo settings
☒	☐	For hierarchical and complex designs, identification of the appropriate level for tests and full reporting of outcomes
☒	☐	Estimates of effect sizes (e.g. Cohen's *d*, Pearson's *r*), indicating how they were calculated
☐	☒	Clearly defined error bars<br>*State explicitly what error bars represent (e.g. SD, SE, CI)*

Our web collection on statistics for biologists may be useful.

Software and code

Policy information about availability of computer code

Data collection	Leica Application Suite X (2.0)
Data analysis	Excel, GraphPad Prism 5, APE A plasmid editor (plasmid assembly), Target Finder : http://crispr.mit.edu/ (Feng Zhang lab) (CRISPR guide)

For manuscripts utilizing custom algorithms or software that are central to the research but not yet described in published literature, software must be made available to editors/reviewers upon request. We strongly encourage code deposition in a community repository (e.g. GitHub). See the Nature Research guidelines for submitting code & software for further information.

Data

Policy information about availability of data

All manuscripts must include a data availability statement. This statement should provide the following information, where applicable:

- Accession codes, unique identifiers, or web links for publicly available datasets
- A list of figures that have associated raw data
- A description of any restrictions on data availability

All materials are fully available.

Field-specific reporting

Please select the best fit for your research. If you are not sure, read the appropriate sections before making your selection.

☒ Life sciences ☐ Behavioural & social sciences ☐ Ecological, evolutionary & environmental sciences

For a reference copy of the document with all sections, see nature.com/authors/policies/ReportingSummary-flat.pdf

Life sciences study design

All studies must disclose on these points even when the disclosure is negative.

Sample size	Statistical methods were not used to determine sample sizes.
Data exclusions	no data were excluded
Replication	Exeriments were performed in duplicate and reproduced three times to assess reproducibility and robustness of each experiment performed.
Randomization	Randomization was not performed since no animals or human participants were used in this study.
Blinding	No blinding was required since randomized group allocation was not performed in this study.

Reporting for specific materials, systems and methods

Materials & experimental systems

n/a	Involved in the study	
☒	☐	Unique biological materials
☐	☒	Antibodies
☐	☒	Eukaryotic cell lines
☒	☐	Palaeontology
☒	☐	Animals and other organisms
☒	☐	Human research participants

Methods

n/a	Involved in the study	
☒	☐	ChIP-seq
☒	☐	Flow cytometry
☒	☐	MRI-based neuroimaging

Antibodies

Antibodies used	The following monoclonal antibodies (mAb) or polyclonal antibodies were used for immunofluorescence labeling or immunoblotting: polyclonal anti-ezrin rabbit antibody (generously provided by Dr. P Mangeat CNRS, UMR5539, Montpellier, France). Goat secondary antibodies were used for immunofluorescence labeling or immunoblotting: anti-mouse IgG (H+L) and anti-rabbit IgG (H+L) coupled to Horse Radish Peroxidase (Jackson ImmunoResearch Laboratories) and anti-mouse IgG (H+L) and anti-rabbit IgG (H+L) coupled to Alexa-488 or Alexa-546 or Alexa-633 (Invitrogen). Mab Anti HA-Tb cryptate and Mab Anti 6HIS-d2 (Cisbio)
Validation	Anti-Ezrin antibody was provided by western blot and immunofluorescence by Paul Mangeat (CNRS UMR5539, Montpellier, France) and described in J Cell Biol. 1989 Mar;108(3):921-30.<br>Secondary antobodies were certified by the manufacturer.

Eukaryotic cell lines

Policy information about cell lines

Cell line source(s)	HEK293 cells were obtained from ATCC bioressource center (#CRL-1573; LGC Standards S.a.r.l., Molsheim, France).<br>hCMEC/D3 cells were provided by Pierre Olivier COURAUD (Cochin Institute, Paris, France).<br>BHK cells were obtained from ATCC bioressource center (#PTA-3544; LGC Standards S.a.r.l., Molsheim, France).<br>C166 cells were obtained from ATCC bioressource center (#CRL-2581; LGC Standards S.a.r.l., Molsheim, France).

Authentication	HEK293 and BHK and C166 cells were authenticated by the supplier. HCMEC/D3 cells were authenticated by us on the basis of cell morpholy and expression of endothelial markers.
Mycoplasma contamination	Cells were routinely tested for mycoplasma contamination by PCR (Takara PCR Mycoplasma Detection kit)
Commonly misidentified lines (See ICLAC register)	HEK293, C166 and BHK cells were obtained from tha ATCC and maintained through a tracable source. No Hela cells (commonly mistaken as HEK cells) were grown in the laboratory.
